# Role of Preoperative Assessment in Predicting Tumor-Induced Plasticity in Patients with Diffuse Gliomas

**DOI:** 10.3390/jcm10051108

**Published:** 2021-03-07

**Authors:** Francesco Latini, Hans Axelson, Markus Fahlström, Malin Jemstedt, Åsa Alberius Munkhammar, Maria Zetterling, Mats Ryttlefors

**Affiliations:** 1 Section of Neurosurgery, Department of Neuroscience, Uppsala University, 75185 Uppsala, Sweden; maria@niklaszetterling.com (M.Z.); mats.ryttlefors@akademiska.se (M.R.); 2 Section of Clinical Neurophysiology, Department of Neuroscience, Uppsala University, 75185 Uppsala, Sweden; hans.axelson@akademiska.se; 3 Section of Radiology, Department of Surgical Sciences, Uppsala University, 75185 Uppsala, Sweden; markus.fahlstrom@radiol.uu.se; 4 Department of Neuroscience, Speech-Language Pathology, Uppsala University, 75185 Uppsala, Sweden; malin.jemstedt@akademiska.se; 5 Rehabilitation and Pain Centre, Uppsala University Hospital, 75185 Uppsala, Sweden; asa.munkhammar@alberius.se

**Keywords:** diffuse gliomas, eloquent tumors, awake surgery, neuropsychological assessment, language assessment, epilepsy, Brain-Grid

## Abstract

When diffuse gliomas (DG) affect the brain’s potential to reorganize functional networks, patients can exhibit seizures and/or language/cognitive impairment. The tumor–brain interaction and the individual connectomic organization cannot be predicted preoperatively. We aimed to, first, investigate the relationship between preoperative assessment and intraoperative findings of eloquent tumors in 36 DG operated with awake surgery. Second, we also studied possible mechanisms of tumor-induced brain reorganization in these patients. FLAIR-MRI sequences were used for tumor volume segmentation and the Brain-Grid system (BG) was used as an overlay for infiltration analysis. Neuropsychological (NPS) and/or language assessments were performed in all patients. The distance between eloquent spots and tumor margins was measured. All variables were used for correlation and logistic regression analyses. Eloquent tumors were detected in 75% of the patients with no single variable able to predict this finding. Impaired NPS functions correlated with invasive tumors, crucial location (A4C2S2/A3C2S2-voxels, left opercular-insular/sub-insular region) and higher risk of eloquent tumors. Epilepsy was correlated with larger tumor volumes and infiltrated A4C2S2/A3C2S2 voxels. Language impairment was correlated with infiltrated A3C2S2 voxel. Peritumoral cortical eloquent spots reflected an early compensative mechanism with age as possible influencing factor. Preoperative NPS impairment is linked with high risk of eloquent tumors. A systematic integration of extensive cognitive assessment and advanced neuroimaging can improve our comprehension of the connectomic brain organization at the individual scale and lead to a better oncological/functional balance.

## 1. Introduction

Recent advances in neuroimaging and direct brain mapping have shown that the brain is capable of significant redistribution of function in response to injury [1,2,3]. Brain plasticity most commonly refers to adaptive changes in neural pathways, synapses and glial cells, leading to functional or morphological reorganization [2,4,5]. Diffuse gliomas (DG) (WHO II and III) are primary slow-growing brain tumors derived from glial cells. Due to their relatively slow natural course, the brain has time to recruit significant compensatory mechanisms to maintain function [2,6,7,8]. Recruitment of healthy/redundant neural circuitry both ipsilateral (first) and contralateral (long term) is a known key mechanism compensating for glioma-induced injury at both cortical and subcortical level [2,4,7,8]. However, age, tumor kinetics, tumor location and early rehabilitation are among the most important factors influencing the neuroplasticity potential of the brain [2,4,6,7]. Hence, tumor lesions that occur in “eloquent” areas, such as Broca’s or Wernicke’s area, may not result in detectable language deficits [9,10,11]. In fact, there have been several reports of resection of presumed critical speech areas, motor areas, visual areas and areas important for cognitive functions on both dominant and non-dominant hemispheres resected in glioma patients [11,12,13,14,15,16,17,18]. On the other hand, there is growing evidence that loss of neuronal network integrity in patients with brain tumors has a negative impact on cerebral function and decreases the threshold to develop seizures [2,4]. Modeling studies in low-grade glioma patients suggested that, when plasticity potential is exhausted, patients can exhibit seizure activity [19,20]. Numerous surgical studies suggest that DG induce brain plasticity through functional compensation and reorganization of the cortex [5,8]. The role of the peritumoral tissue seems strongly related to the presenting symptoms. Electrophysiological recordings and histopathological analyses support this hypothesis by demonstrating that epileptic seizures arise from the peritumoral neocortex and not from the tumor core and that infiltrated isolated glioma cells permeate the peritumoral neocortex [21,22,23,24,25,26]. In addition to cortical gray matter plasticity, the subcortical pathways play a crucial role in shaping cortical reorganization [19]. DG migrate along the white matter pathways with an invasion rate estimated to be about five times higher than in the gray matter [19,27]. The continuous expansion of DG represents an important factor influencing neuroplasticity and then the surgical result because of the so-called minimal common brain and the lower subcortical plasticity [28,29]. Patients with DG in eloquent areas invading subcortical pathways may be at risk for neurological impairment already at the time of radiological diagnosis. Hence, the infiltration of white matter represents a very important factor in the management of DG. To better classify the invasiveness of DG, a novel radiological observational tool has been proposed: The Brain-Grid [30,31]. This tool includes white matter infiltration in the standard topographical classification of DG and is used to identify differences in preferential locations and infiltration pathways in DG subtypes [31]. However, despite advances in diagnostic methods and surgical techniques, allowing extensive and safe tumor resections as well as the introduction of molecular tumor markers guiding therapeutic decisions, the clinical management of DG remains challenging [32,33,34,35]. Advanced neuroimaging techniques such as functional MRI and DTI are not able to differentiate essential cortical/subcortical areas (which should be surgically preserved) from the “modulatory” areas that can be functionally compensated and resected without inducing permanent deficits [10,36,37,38,39]. In addition, functional modifications induced by tumor growing patterns at both cortical and axonal levels [40] encourage the study of the brain functional organization and connectivity at individual level. The aim is to both select the best indications for surgery and perform a resection with the optimal benefit/risk ratio [28,29]. Surgical resection with direct electrical stimulation (DES) and functional mapping of language and high-order functions remain the gold standard technique for these patients to detect and save functional epicenters [41,42,43]. In fact, the risk of residual tumor depends on the presence within tumor area of functional networks not yet reshaped or compensated and therefore represents an important indirect index of neuroplasticity [4,42]. The complex interactions between the tumor and the host brain are still not fully understood and a prediction of the individual connectomic organization for each patient remains impossible to predict preoperatively.

We had two main aims with this study. The first aim was to investigate whether a correlation exists between clinical variables at the moment of radiological diagnosis (symptoms onset, neuropsychological impairment or language impairment) and intraoperative findings of eloquent tumor from brain mapping.

Then, analyzing all the eloquent spots from cortical and subcortical mapping, we aimed to identify the relationship between tumor extension and the presence of eloquent spots (anatomical sites positive at the DES) within the tumorous tissue, possibly reflecting mechanisms of local tumor-induced plasticity/reorganization.

## 2. Materials and Methods

### 2.1. Patient Selection

Patients (>18 years) presenting with a radiological diagnosis of suspected low-grade glioma were consecutively recruited at the Department of Neurosurgery, Uppsala University-Hospital, Uppsala, Sweden, and enrolled in the study between August 2014 and August 2020. Exclusion criteria for this study were previous resection for brain tumors, previous radio-chemotherapy, severe respiratory diseases, history of psychiatric diseases or psychiatric contraindication, severe language impairment. The study was approved by the institutional ethics review board (2015/210/2). Informed consent was obtained prior to surgery at the Department of Neurosurgery, Uppsala University Hospital.

### 2.2. Imaging

MRI including tractography and neuronavigation sequences was done prior to surgery and postoperative MRI within 48 h and 3 months after surgery. A conventional MRI protocol consisting of T2W, T2-FLAIR (in low slice thickness, 1 mm), diffusion sequences and pre- and post-contrast T1w were acquired according to our standard glioma imaging practice [44,45]. Morphological MRI sequences (volumetric T1W, T2W and T2-FLAIR) were used to assess brain tumor location and heterogeneity, mass effect, radiological border (sharp or diffuse), contrast enhancement and the presence of multiple brain lesions [44,45,46]. T2 turbo spin echo or T2 FLAIR images in Vue picture archiving and communication system (PACS) software (version 11.1.0, Carestream Health Inc., Rochester, NY, USA) were used to segment the lesions both pre- and postoperatively with the aid of a semiautomatic method (Livewire Algorithm) [47]

Morphologic and diffusion MRI of the brain was performed on a three tesla (3T) MRI scanner (Philips Achieva, Best, The Netherlands). DTI was performed using a single-shot spin echo sequence with echo-planar imaging, 60 contiguous slices, voxel size 2 × 2 × 2 mm^3^, Echo time/Repetition time of 77/6626 ms/ms, a diffusion-weighting factor b = 1000 s/mm^2^ and diffusion encoding along 48 directions. Motion and eddy current correction of acquired DTI data was automatically performed in BrainEx (version 2.3.6. NordicNeuroLab AS, Bergen, Norway). The parametric maps of Fractional anisotropy, Axial diffusivity and Radial diffusivity were calculated and merged on T2-FLAIR volumetric sequences. Streamline tractography was performed with a fractional anisotropy threshold of 0.1, an angular threshold of 45° and minimum length of 20 mm. BrainEx was used for the placement and drawing of regions of interest (ROIs) and regions of avoidance (ROAs). The anatomical placement of the ROIs and ROAs was manually performed using the most validated DTI atlases as references [48,49,50]. Using a two ROIs approach, inferior occipitofrontal fasciculus (IFOF), superior longitudinal fasciculus (both horizontal indirect component hSLF and vertical indirect component vSLF), arcuate fasciculus (AF), cortico-spinal tract (CST), frontal aslant tract (FAT) and optic radiation (OR) were reconstructed in each patient on the interested hemisphere.

MRI sequences and tractography results were transferred to Brainlab (Brainlab, Munich, Germany) server and uploaded into the neuronavigation system and preoperative planning software. Intraoperatively, each of the positive functional points detected subcortical STS was acquired with neuronavigation for the postoperative analysis (see below).

### 2.3. Brain-Grid Analysis

**Figure 1:**
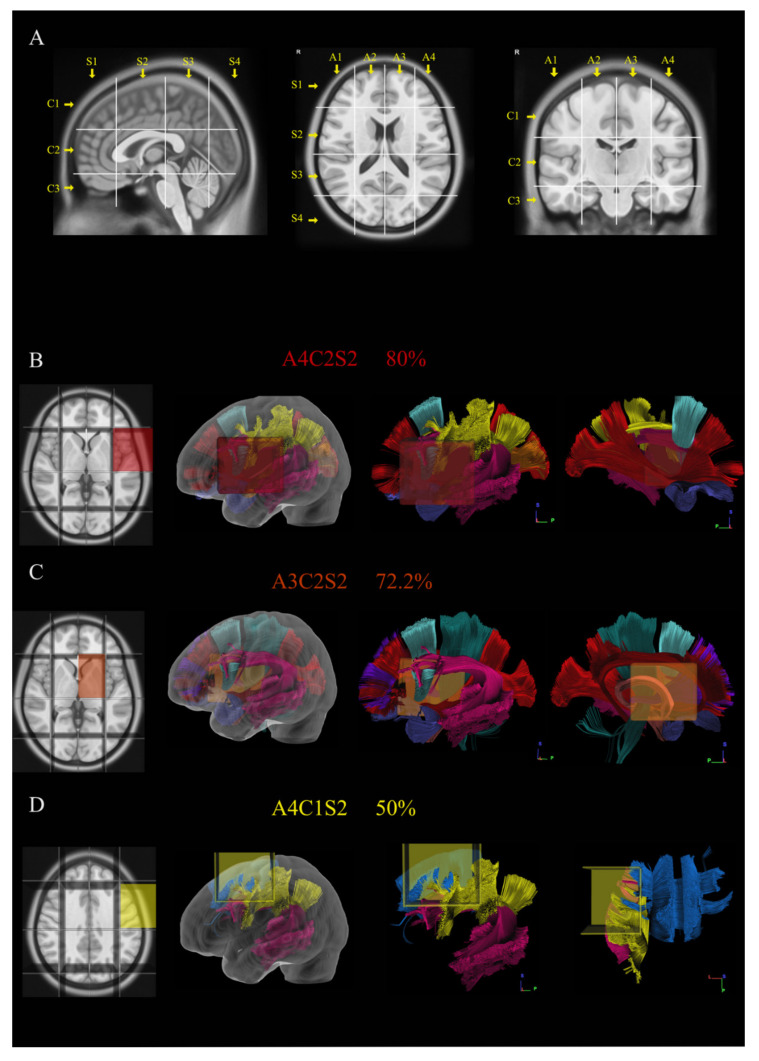
The picture shows in (**A**) the construction and use of Brain-Grid system in MNI space. Three sagittal lines cross the anterior insular point (the most anterior landmark of the insular sulcus), the posterior insular point and the temporo-occipital junction (between the posterior portion of the fusiform gyrus and the inferior occipital sulcus more basally on the axial plane). These lines segment the whole brain into four grid voxels labeled with the first coordinate S (from sagittal line). The S1 voxel is the pre-insular/prefrontal portion of both hemispheres. The S2 is enclosed within the anterior insular point and posterior insular point (landmark for the second sagittal line). The S3 includes the retro-insular region and the parietal lobe, and the S4 includes primarily the occipital lobe and the border with the parieto-occipital sulcus. On the coronal plane, two parallel lines cross the inferior insular point (the lowest limit of the insular sulcus), the floor of the third ventricle and the mammillary bodies, while the second line passes through the cistern/space between the Cingular gyrus and the callosal body in the midline. Three voxels are created and named after the coordinate C (from coronal plane) with C1, which is the supra callosal; C2 between the corpus callosum and the mammillary bodies; and C3, which includes the region of temporal lobe, occipital lobes and brainstem/cerebellum under the mammillary bodies. On the axial slices, the middle frontal sulcus bilaterally and the midline are chosen as three landmarks for three parallel lines. In this way, four longitudinal segments are created, termed A1–A4, from the right lateral side to the left lateral side. In total, 48 Brain-Grid voxels are created by the intersection of three sagittal lines, two coronal lines and three axial lines. (**B**–**D**) Three-dimensional (3D) reconstructions of the three most infiltrated BG voxels and the reconstruction of white matter bundles according to the BG atlas [24]: (**B**) A4C2S2 was infiltrated in 80% of the cases in our population. From the right to the left: position of the BG-voxel on axial T1MR sequence with morphological details; 3D glass left cerebral hemisphere with the position of the BG voxel; and the 3D reconstruction of the major white matter bundles included in the BG voxel. The glass hemisphere has been removed to show the BG voxel only with the white matter bundles from lateral sagittal view of the left hemisphere. The voxel included fibers of the indirect segment of the superior longitudinal fasciculus (hSLF; in yellow), arcuate fasciculus (AF; pink); frontal aslant tract (FAT; turquoise), anterior temporal termination of the middle longitudinal fasciculus (MdLF; orange), uncinate fasciculus (UF; orchid) and anterior termination of the inferior occipito frontal fasciculus (IFOF; red). The last image shows the reconstruction of the same voxel from the medial sagittal perspective. (**C**) A3C2S2 was infiltrated in 72.2% of the cases in our population. From the right to the left: position of the BG-voxel on axial T1MR sequence with morphological details; 3D glass left cerebral hemisphere with the position of the BG voxel; and the 3D reconstruction of the major white matter bundles included in the BG voxel. The glass hemisphere has been removed to show the BG-voxel only with the white matter bundles from lateral sagittal view of the left hemisphere. The voxel included fibers of AF (pink), FAT (turquoise), UF (orchid) and IFOF (red). The last image shows the reconstruction of the same voxel from the medial sagittal perspective to show the involvement of anterior thalamic radiation (ATR; grape), fornix (Fo; salmon), cingulum (Ci; cayenne) and Cortico-spinal tract (CST; teal). (**D**) A4C1S2 was infiltrated in 50% of the cases in our population. From the right to the left: position of the BG-voxel on axial T1MR sequence with morphological details; 3D glass left cerebral hemisphere with the position of the BG voxel; and the 3D reconstruction of the major white matter bundles included in the BG voxel. The glass hemisphere has been removed to show the BG-voxel only with the white matter bundles from lateral sagittal view of the left hemisphere. The voxel included fibers of hSLF (yellow), AF (pink) and fibers from the body of the corpus callosum (CCb; aqua). The last image shows the reconstruction of the same voxel from the dorsal perspective.

A standardized grid created by intersecting longitudinal lines on the axial, sagittal and coronal planes was reconstructed within MNI space (Figure 1A) as previously described [30,31] (a detailed description of the methods is provided in the Figure 1 legend). Applying the Brain-Grid classification system using the DSI studio in Montreal neurological Institute (MNI) space, the number of voxels involved by tumor lesion was recorded. The tumor lesions were reclassified according to the Brain-Grid system to obtain quantitative (number of BG voxels and frequency of infiltration) and qualitative data (preferential localization).

### 2.4. Language and Neuropsychological Evaluation

Patients were assessed by a speech therapist and a neuropsychologist before surgery. In addition, three- and twelve-months postoperative evaluations are included in our standard protocol for awake surgery but not reported here. The linguistic evaluation contained confrontation naming, language comprehension, phonological and semantic word fluency, tests of reading and writing and phonological ability. The neuropsychological assessment contained tests of attention and working memory, processing speed, learning and long-term memory (verbal as well as visual), visuospatial construction, executive functioning and self-reported anxiety and depression. (see File S1 for the test choices).

### 2.5. Surgical and Stimulation Technique

The anesthetic technique was according to an asleep–awake–sedation/asleep protocol. The surgical resection aimed to reach functional limits and/or crucial anatomical structures such as basal ganglia or anterior perforate substance. For the cortical and subcortical mapping, we utilized a combination of bipolar and monopolar cortical-subcortical stimulation, as described by other studies [51,52,53]. Cortical electrical stimulation (60 Hz, biphasic pulses with a 1 ms duration for 3 s) was performed using a bipolar probe with an interelectrode distance of 5 mm (Dr. Langer Medical GmbH, Waldkirch, Germany) according to Penfield stimulation technique (PS) [54]. The required stimulation intensity for cortical mapping was established by stimulating either the ventral premotor or primary motor cortex while observing corresponding clinical effects (i.e., speech arrest during counting or tonic muscle contractions, respectively). The corticectomy started after the cortical mapping and the resection of subcortical structures was continuous until functional limits were detected leaving the pathological tissue in situ. This reduced the possibility to anatomical shift between the cortical and subcortical eloquent points.

The PS intensity was the same for cortical and subsequent subcortical stimulation. Language related tests (e.g., picture naming, reading or repetition) were performed by a speech therapist or a neuropsychologist during electrical stimulation. A custom-made system was used for intraoperative testing of language and other cognitive function. Language test-related images were displayed on a PC monitor and presented to the patient at a constant pace. The electrical stimulation was controlled from a neuromonitoring device (Cadwell Industries, Washington, DC, USA) that also monitored cortical EEG, free-running EMG and motor evoked potential (MEP). Stimulus-induced muscle contractions were also monitored by clinical inspection. MEP recordings were used for subcortical motor mapping. Cortical EEG was recorded from two separate one-by-four strip electrodes (Ad Tech Medical Instruments, Wisconsin, DC, USA) to detect seizure activity or after-discharges. Stimulation results/effects were documented intraoperatively and also video captured for postoperative review.

At the subcortical level, we also used continuous (3 Hz) cathodal short-train stimulation (STS) (5 monophasic pulses, 4 ms interpulse interval and 0.5 ms pulse duration) which was delivered via the tip of a specially designed suction probe (Inomed, Emmendingen, Germany). Language interference and other clinical effects from STS were compared with those produced by PS (feasibility and reliability of continuous STS are explored in an ongoing separate study). Anatomical sites that were positive at PS (cortical or subcortical) or consistently positive at 5 mA with STS (subcortical) were considered eloquent and acquired on the neuronavigation system. After registration of the eloquent spots the rest of the tumor was resected with ultrasonic dissector to reveal the medial or deep functional limit of the resection. In the case of brain-shift, the intraoperative navigated ultrasound (Flex focus 800, BK Medical, Denmark) probe was used to adjust the navigation accuracy, as described by other authors [55,56,57].

### 2.6. Postoperative Analysis of Eloquent Points

The linear distance between the 3D defined eloquent points acquired intraoperatively and tumor margins were measured postoperatively (Brainlab software). Tumors containing eloquent points within areas defined by hyperintensity on FLAIR sequences (cortical or subcortical) were considered eloquent. Eloquent points within 5–10 mm from the FLAIR signal margin were considered peritumoral, while those acquired beyond 10 mm from the FLAIR signal were considered outside the tumor area. Pre- and postoperative images (acquired within 48 h and 3 months after the operation) were merged to detect and spatially locate the presence of eventual residual tumor.

### 2.7. Statistical Analysis

For descriptive analysis, mean values and standard deviation (SD) were calculated for age, volume, extent of resection, survival from diagnosis and the number of eloquent spots detected intraoperatively at the cortical and subcortical level. Median and interquartile range (IQR) were calculated for the number of Brain-Grid voxels. Total values and percentage were calculated for gender, epileptic onset, eloquent tumors, preoperative neuropsychological or language impairment. Group comparison between eloquent and non-eloquent tumors and tumor subtypes and histology were performed with Mann–Whitney U test and Kruskal–Wallis test for all variables analyzed. A Shapiro–Wilk test was used to test normal distribution of the continuous variables. A Spearman correlation analysis was chosen for the more relevant continuous variables (age, tumor volume, BG-voxels, number of eloquent points cortical/subcortical, intratumoral/peritumoral and resection grade). For age, number of BG-voxels and tumor volume, an optimal cut-off choice in two groups was made according to receiver operating characteristics curves (ROC) to convert them in dichotomous variables. Pearson’s Chi-square test and contingency test were used for groups correlation for categorical and dichotomous variables (gender, age cut-off, epileptic onset, preoperative NPS and language impairment, eloquent tumors, radiological border, volume cut-off, number of BG-voxels cut-off, histology and tumor grade). A group comparison between younger patients and older patients was performed post-hoc with Mann–Whitney test for the following variables: histology, tumor grade, tumor volume, tumor location, BG voxels, clinical variables and intraoperative variables including the number of eloquent spots.

We used binary logistic regression model to investigate the relationship between clinical variables (epileptic onset, NPS impairment and language impairment) considered as dependent variables, the intraoperative variables (eloquent spots, cortical, subcortical, intratumoral and peritumoral) and the most often infiltrated BG voxels. Finally, we used a multivariate binary logistic regression model to identify independent predictors of eloquent tumors. Forward step-wise proportional hazards modeling was performed to assess the relative and independent prognostic capacity of each parameter. All statistical analyses were performed at a significance level of *p* < 0.05 and Confidence Interval of 95%, using the statistical package SPSS 25.0 (SPSS, Inc., Chicago, IL, USA).

## 3. Results

### 3.1. Patient Population

Thirty-six patients were enrolled in this study. In 34 patients, the tumors were located on the left dominant hemisphere, while, in two patients, the tumor was located on the right hemisphere but the patients displayed preoperative bi-hemispheric dominance. The clinical onset included epileptic symptoms in 72.3% of the patients. The preoperative language examination revealed that 66.7% of the patients suffered some form of speech impairment (semantic 38.9%, phonology 13.9%, comprehension 8.3%, dysarthria 5.3% and verbal memory 2.8%), while 84.6% of the analyzed patients displayed neuropsychological impairment (working memory 53.3%, attention 40%, learning 26.6%, executive functions 26.6% and memory 20%). A summary of demographic, histological and clinical information is displayed in Table 1.

### 3.2. Radiological Features

The radiological border was diffuse in 61% of the cases. In 55.6% of patients, the tumor volume was larger than 56 mL (mean value/cutoff value according to ROC curves). A summary of radiological information is displayed in Table 1. The Brain-Grid classification system was applied in all MRI scans. The quantitative analysis showed a median of six BG voxels infiltrated at the radiological diagnosis. Overall, 55.6% of patients displayed more than six BG voxels. The qualitative analysis demonstrated that the A4C2S2 (left fronto-temporal opercula and insular cortex) had the highest rate of invasion (80%), followed by A3C2S2 (Left subcortical insular and basal ganglia) (72.2%) and A4C1S2 (left dorsolateral prefrontal cortex) (50%). The position and the relationship with the major white matter bundles for the infiltrated BG-voxels is displayed in Figure 1B–D.

### 3.3. Intraoperative Findings

We found intratumoral eloquent spots in 75% of the cases. The mean resection rate was 79% (SD 15.8). In total, 254 positive responses (156 cortical, 98 subcortical) were collected by using direct cortical or subcortical stimulation among 11 cortical and 13 subcortical functional domains. A summary of surgical information is displayed in Table 1 and Table 2. The cortical functional responses collected included: motor, sensory, motor control (including negative motor mapping), spatial perception, speech articulation domain (including verbal apraxia, latency and dysarthria), speech output domain (speech arrest), anomia, phonological paraphasia, auditory phenomena, reading and working memory.

Subcortical functional responses included: motor, sensory, motor control (including negative motor mapping), spatial perception, speech articulation domain (including verbal apraxia, latency and dysarthria), speech output domain (speech arrest), anomia, phonological paraphasia, auditory phenomena, reading, working memory, visual field and visceral phenomena.

### 3.4. Postoperative Analysis

**Figure 2:**
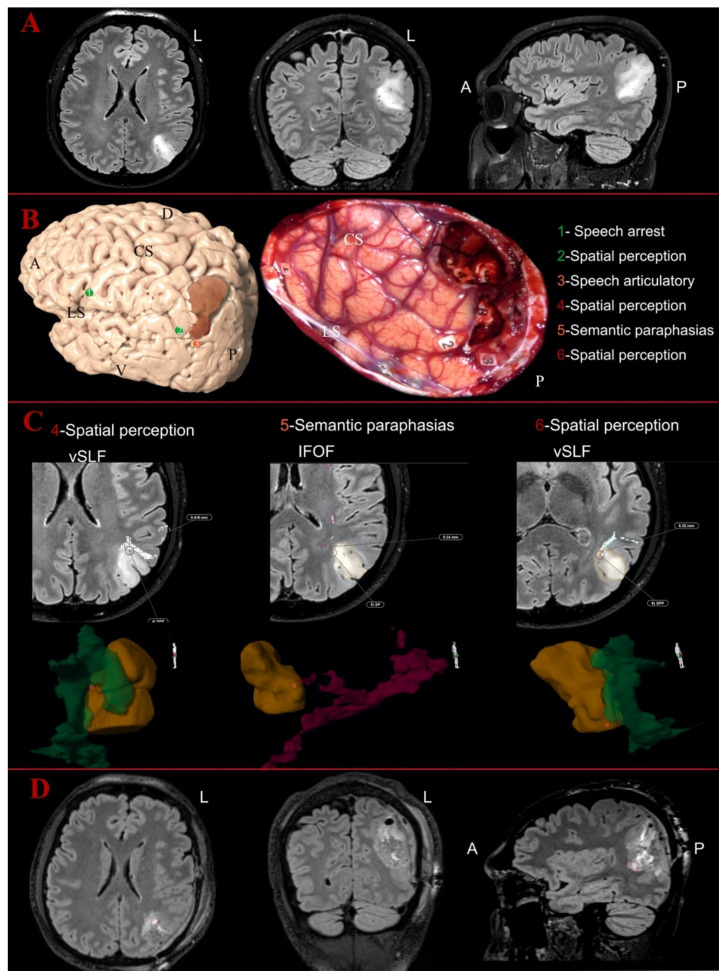
Illustrative case of parietal Oligodendroglioma Grade III on the left hemisphere: (**A**) the preoperative MR images of FLAIR sequences in axial, coronal and sagittal perspective; and (**B**) the 3D reconstruction of the left hemisphere with the tumor (in dark orange) and the distribution of the cortical eloquent spots in the upper part. The middle part shows the intraoperative picture after the mapping and the resection showing the distribution of the cortical and subcortical eloquent spots, listed in the lower part of the image. The spots are listed in green if considered outside the tumor area, in orange if peritumoral (<10 mm) or in red if considered intratumoral. (**C**) The postoperative analysis of the subcortical spots displayed in axial slices with the distance from the related white matter bundles and the 3D reconstruction of the points-tumor and white matter reconstructions. The vertical segment of superior longitudinal fasciculus is displayed in dark green, while the inferior fronto-occipital fasciculus (IFOF) is in magenta here. Number 4 and Number 6 were detected as intratumoral eloquent spots eliciting spatial perception disturbances, but they were not inside the vSLF reconstruction. Spot Number 5 (elicited sematic paraphasia) was detected as peritumoral and again not inside the course of the IFOF according to the 3D tractography reconstruction. (**D**) The postoperative images with FLAIR sequences in axial, coronal and sagittal perspective, showing in axial and sagittal a portion of the residual tumor in the ventral and anterior portion of the surgical cavity. A, anterior; D, dorsal; L, left; V, ventral; P, posterior; CS, central sulcus; LS, lateral sulcus.

**Figure 3:**
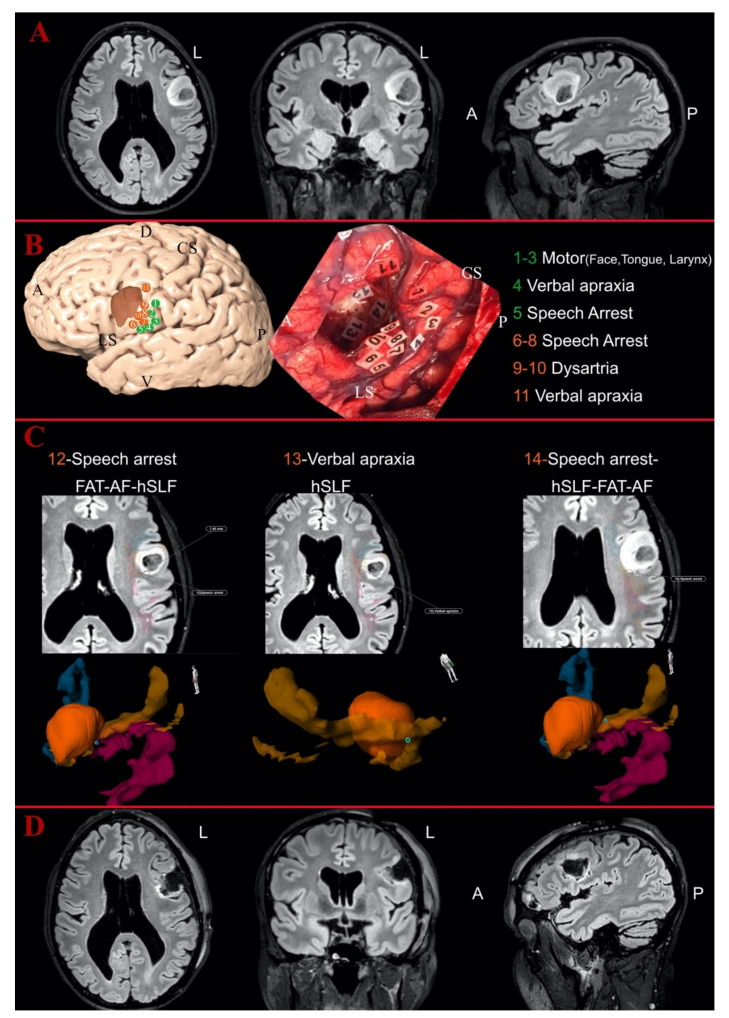
Illustrative case of fronto-opercular Astrocytoma Grade II on the left side: (**A**) the preoperative MR images of FLAIR sequences in axial, coronal and sagittal perspective; and (**B**) the 3D reconstruction of the left hemisphere with the tumor (in dark orange) and the distribution of the cortical eloquent spots in the upper part. The middle part shows the intraoperative picture after the mapping and the resection showing the distribution of the cortical and subcortical eloquent spots, listed in the lower part of the image. The spots are listed in green if considered outside the tumor area, in orange if peritumoral (<10 mm) or in red if considered intratumoral. (**C**) The postoperative analysis of the subcortical spots displayed in axial slices with the distance from the related white matter bundles and the 3D reconstruction of the points-tumor and white matter reconstructions. Horizontal segment of superior longitudinal fasciculus (orange), FAT (blue) and AF (magenta). Spot Numbers 12–14 were detected as peritumoral eloquent spots, eliciting, respectively, speech arrest, verbal apraxia and speech arrest, but they were not inside the vSLF reconstruction. Spot Number 5 (elicited sematic paraphasia) was detected as peritumoral and again not inside the course of the IFOF according to the 3D tractography reconstruction. The position of Number 12 did not match any of the related white matter bundles for speech arrest, while Numbers 13 and 14 were located along the course of the hSLF. (**D**) The postoperative images with FLAIR sequences in axial, coronal and sagittal perspective, showing no residual tumor in the medial portion of the surgical cavity but only postoperative signal changes. A, anterior; D, dorsal; L, left; V, ventral; P, posterior; CS, central sulcus; LS, lateral sulcus.

We acquired 61 intratumoral and 85 peritumoral eloquent spots. In total, 108 eloquent spots were detected outside the peritumoral area. All intraoperative eloquent spots according to the tumor locations for each patient are displayed in Table 2. The eloquent points were matched with the supposed white matter bundle supporting that function as described in other studies [7,29,59]. The position of the eloquent spots and their relationship with tumors and white matter bundles are displayed in Figure 2 and Figure 3. A close proximity of eloquent spots with the reconstructed white matter bundles (<5 mm) was detected in 40% of the cortical and 50% of the subcortical points. Two illustrative cases showing the mapping results are displayed in Figure 2 and Figure 3.

### 3.5. Statistical Results

In the comparison between eloquent and non-eloquent tumors, no difference was detected for age, gender, tumor grade, histology, tumor volume, number of BG-voxels, radiological border, epileptic onset, preoperative language or neuropsychological impairment.

In the correlation analysis, older age negatively correlated with the number of peritumoral cortical eloquent spots (*p* < 0.05) and directly correlated with the intratumoral cortical eloquent spots (*p* < 0.05). Preoperative tumor volume was directly correlated with the number of BG voxels (*p* < 0.001) with epileptic onset (*p* < 0.05) and negatively correlated with the number peritumoral subcortical eloquent spots (*p* < 0.05) and with the resection grade (*p* < 0.05). The extent of resection was positively correlated with the number of peritumoral eloquent spots subcortical (*p* < 0.05) and negatively correlated with the number of intratumoral eloquent spots cortical (*p* < 0.05). The number of intratumoral cortical eloquent spots was negatively correlated with the number of peritumoral cortical eloquent spots (*p* < 0.05). Epileptic onset was correlated with tumor volume (*p* < 0.05) and A3C2S2/A4C2S2 voxels infiltration (*p* < 0.05). Preoperative NPS impairment was correlated with the number of BG voxels (>6) (*p* < 0.05). The complete correlation analyses are displayed in Table 3 and Table 4.

Group comparison between younger and older patients (cut-off at 38 years old) showed no significative difference for gender, histology, tumor grade, radiological borders, tumor volume, number of BG voxels, clinical variables or location (*p* > 0.05). The two groups displayed a significative difference in the number of intratumoral cortical eloquent spots (*p* < 0.05) and the number of peritumoral cortical eloquent spots (*p* < 0.001).

In the univariate logistic regression analysis, the presence of intratumoral eloquent spots at the subcortical level was correlated with a higher risk for NPS impairment (p< 0.05) and epilepsy (*p* < 0.05). A slightly higher risk of epileptic onset was also correlated with the presence of peritumoral eloquent spots at the cortical level (*p* < 0.05). The infiltration of the BG voxel A3C2S2 (left subcortical insular and basal ganglia) was correlated with a significant high risk of NPS impairment (*p* < 0.05, HR 7.5), epilepsy (*p* < 0.01, HR 5.5) and language impairment (*p* < 0.05, HR 1.2). The infiltration of the BG voxel A4C2S2 (left fronto-temporal opercula and insular cortex) was correlated with a significant higher risk of NPS impairment (*p* < 0.05, HR 3.7) and epileptic onset (*p* < 0.01, HR 6.2).

In the multivariate logistic regression analysis, preoperative NPS impairment was the only independent variable able to predict the intraoperative finding of eloquent tumors (*p* < 0.01, HR 6.3). A summary of the significative results from the logistic regression analysis is displayed in Table 5.

## 4. Discussion

Our study showed three main results. First, the preoperative evidence of neuropsychological impairment was linked with a high risk of finding an eloquent tumor at the brain mapping. Second, we found correlations between radiological/topographical features and clinical variables. Third, our results from intraoperative mapping suggested a pattern of tumor-induced changes in the peritumoral functional environment.

### 4.1. Preoperative NPS Assessment Was Linked with Intraoperative Findings

In our population, 84.6% of the patients displayed neuropsychological symptoms in the preoperative assessment. In 75% of patients, there were intraoperative findings of eloquent spots. The presence of preoperative neuropsychological symptoms was the only independent factor linked to a high risk (HR 6.3) of finding an eloquent tumor intraoperatively. We were unable to detect any other differences between eloquent and non-eloquent tumors in the remaining preoperative variables analyzed (epidemiological, radiological, histological or clinical). This may reflect the already known difficulties in predicting the functional outcomes of patients with low-grade gliomas without brain mapping [36,41]. Considering the preferential locations of these lesions and the white matter structures involved, the use of intraoperative mapping is now mandatory to preserve high-order functions and achieve an oncological–functional balance in low-grade gliomas [31,41,59,60,61]. A careful preoperative assessment seems of paramount importance to identify good candidates for the surgery and tailor brain mapping at the individual scale [20,62,63]. Several studies suggest that the local growth of gliomas affect the global brain functional organization by causing impaired communication of large-scale networks for cognition and behavior [2,64]. The insult to the subcortical white matter structural connectivity is correlated with a decline in cognitive functions, confirming that axonal bundles represent a major limitation in neuroplasticity [64,65]. Moreover, the link between global network disturbance and a higher risk of finding eloquent spots within the tumor area may suggest that an exhaustion of local adaptation and the insult to global redistribution of the neural activity may be closely linked in time, at the moment of radiological diagnosis. fMRI studies demonstrated that patients with low-grade gliomas may need to activate multiple brain areas to perform a required task because of the reduced global default mode network efficiency [66,67]. Neuropsychological impairment may emerge when the global networks are no longer able to recruit and compensate for the local invasion of large-scale networks. This may be important information to take into account during the preoperative assessment in selecting intraoperative tasks and discussing possible functional outcomes with these patients.

### 4.2. Correlations between Clinical Variables and Radiological/Topographical Features

NPS impairment was found in 84% of our patients. The number of BG-voxels (>6) was indeed correlated with a preoperative cognitive or NPS problem. Based on the intrinsic advantage of BG-voxel analysis in quantifying invasiveness of DG [30,31], this result may suggest a predominant invasion to the subcortical networks as a constant finding in NPS impairment as supported by other authors [2,27,64]. A higher number of BG-voxels usually indicates a possible invasion of interhemispheric and periventricular white matter networks with secondary insult to large-scale networks characterized by less plastic potential [2,64]. In the qualitative analysis of BG-voxels, the infiltration of A3C2S2 voxel was linked with a five times higher risk for the patient to demonstrate NPS preoperative impairment. This region (sub-insular/basal ganglia on the left side) has been often associated with cognitive or psychiatric disturbances [33,68,69,70]. In our population, DG associated with impaired neuropsychological performance at the moment of diagnosis displayed more invasive tumors (>6 BG-voxels), a crucial preferential location (A3C2S2 voxel) and a higher risk of intraoperative findings of eloquent tumors. Altogether, these results support two main considerations. First, we support the role of neuropsychological preoperative assessment as an important part of preoperative clinical assessment in patients with DG [56,62]. NPS assessment may be able to detect differences in invasiveness of brain tumors, clinically detecting damage to large-scale networks. Second, since the evidence of NPS impairment suggests the exhaustion of adaptive mechanisms and the insult of large-scale networks, an early diagnosis and surgical approach may be advocated [12,71,72] to possibly increase the resection before the tumors reach a not compensable level of invasion.

Epileptic onset was the second most frequent clinical variable, displayed in 77.3% of the patients. Epilepsy as onset symptom was correlated with a larger tumor volume (>56 mL). This correlation has not been found in other studies [22,27] and for this reason should be carefully interpreted. Compared with other articles [22,27], the volume computation in our study was performed with a semiautomatic method able to follow the signal hyperintensity even with diffuse border and with good reliability [47,73]. Moreover, we adopted a cut-off of the volumes based on ROC curves and not on the median value, this may have possibly increased the specificity for the epileptic onset. Beyond the methodological consideration, the role of tumor volume as factor affecting the functional status in DG have been extensively discussed in the last years. Some authors suggested that larger tumors are linked with less functional reorganization [2,52], implying that larger gliomas trigger compensatory neuroplasticity before the surgery. There is, however, evidence that the combination between volume and special locations may induce epileptic onset as a multifactorial result [27].

First, in our population, the epileptic onset was correlated with volumes larger than 56 mL but not with the number of BG voxels. The main advantage of using BG voxel analysis as a complement to the standard volume computation is the possibility to acquire information regarding the invasiveness of the tumor through the subcortical regions [30,31]. Despite the expected correlation between tumor volume and BG voxels, our results suggest that the epileptic onset may reflect a local/focal phenomenon. This may be due to the higher volume expansion at the cortical level rather than infiltrative feature of the tumor through subcortical areas, as also supported by other authors [20,27]. In fact, slow-growing tumors could produce an epileptogenic environment by partial deafferentation of cortical regions, thus causing a denervation hypersensitivity [24]. Studies using magnetoencephalographic recordings have shown that functional connectivity and network topology are significantly altered in DG cases. Low-frequency connectivity seems pathologically increased, probably due to an adaptive recruitment, and the normal “small-world network” configuration is altered, leading to a lower threshold for seizures [24,26,74,75,76] in the peritumoral cortical areas [27].

Second, we investigated the topography of tumors inducing epileptic onset with BG-voxel analysis. The infiltration of A4C2S2 and the A3C2S2 voxels increased the risk of epileptic onset almost six times. The left fronto-temporal opercula and the insular/sub-insular/basal ganglia regions seem to represent a crucial location for the epileptic onset in DG patients. These regions are also among the most often infiltrated as preferential location in DG [30,31,77] and often correlated with neurological impairment and epilepsy [22,24,27].

Language impairment was found at the preoperative evaluation in 66.7% of our patients. No correlation with radiological variables (volume, number of BG-voxels or radiological border) was found. The possible risk for language problems were linked to the topographical variables. The infiltration of A3C2S2 BG-voxel was linked with a higher risk of language impairment. This voxel includes the sub-insular/basal ganglia region on the left side, and it is considered highly functional as a part of the minimal common brain [28,29]. This region is among the less plastic ones, due to presence of basal ganglia, fibers from the external and internal capsule and fronto-striatal circuits [28,29,30]. An infiltrative tumor reaching this region would probably affect white matter fibers supporting large networks in their connecting hubs rather than peripheral cortical terminations with a lower possibility for early adaptive mechanisms due to the limited white matter plasticity [2,4,64,78].

### 4.3. Patterns of Tumor-Induced Changes in the Peritumoral White Matter Networks

We investigated the relationship among eloquent spots in the tumoral/peritumoral area to understand if one could possibly predict a pattern of distribution of the eloquent spots. First, as stated above, the presence of eloquent spots inside the tumor area was linked with NPS impairment. This suggests that the insult to large-scale networks may decrease the possibility for the peritumoral environment to compensate at the local level. What we know about the peritumoral milieu is that, when epileptic onset is triggered, it starts from the peritumoral cortical areas and not from the tumor core [2,24,27]. In support of this theory, the epileptic onset was correlated with the presence of peritumoral eloquent spots at the cortical level and at the same time the intratumoral eloquent spots subcortically. This implicates that when the epileptic activity emerges: (1) the tumor has already invaded subcortical larger networks limiting the possible large-scale reorganization/adaptation of the neural activity; (2) the peritumoral cortices have been recruited within the same functional hub through short intermediate fibers as the first mechanism of local reorganization; and (3) the subcortical networks (intermediate/long fibers) newly invaded by the tumors (intratumoral subcortical eloquent spots) are a constant correlation in both epilepsy and NPS impairment.

Furthermore, if we analyze the relationship among the different categories of eloquent spots, we identify a negative correlation between peritumoral cortical eloquent spots and intratumoral cortical eloquent spots. This suggests that the two findings are signs of different stages of the adaptive process during gliomas formation and proliferation that are not often present at the same time [2,42,64]. Interestingly, we found that age was a possible factor affecting this adaptive potential. The two groups displayed a difference in the number of intratumoral cortical and peritumoral cortical eloquent spots. Older age was negatively correlated with the number of peritumoral cortical eloquent spots and directly correlated with the number of intratumoral cortical eloquent spots. This seems in agreement with other authors suggesting a decrease rate of myelin/white matter plasticity with age [6,79]. We may hypothesize that small cortical DG may activate early cortical adaptive mechanism in younger people, who are able to compensate for the gliomas expansion recruiting adjacent cortices. An increase in tumor volume leads to epilepsy due to the insult of large-scale networks, unable to modulate the peritumoral environment. At this point, the invasion of large-scale networks may reflect patients NPS preoperative impairment and the possible exhaustion of adaptive mechanisms at the peritumoral level, due to a lower white matter plasticity.

Finally, no correlation was detected between the number or distribution of cortical spots and the subcortical eloquent spots. In other words, the risk of finding eloquent spots at the subcortical level cannot be predicted by the cortical mapping. For this reason, awake surgery with direct cortical subcortical mapping seems the only technique able to achieve an oncological–functional balance in patients with DG at individual level.

### 4.4. Limitations

Our study has some limitations. The first one regards the size and type of our population. Of all the patients operated in our center with awake surgery, only 36 until now had a diagnosis of DG (WHO-II-III), no previous resection, no previous radio-chemotherapy and at the same time a cognitive and/or linguistic performance suitable for awake surgery. This reduced the potential number of analyses. For instance, no difference was detected among histological four groups, and the majority of the patients displayed a mutated status of IDH1 while in 10 patients this status was not available. Therefore, we considered further analysis based on histology/molecular status beyond the aim of this study and rather speculative with this population. Moreover, since the original indication for awake surgery in our center was the language mapping, only patients with gliomas harboring dominant hemispheres (two bi-hemispheric dominant) were enrolled in this study, influencing the interpretation of the results. Despite these limitations, our study describes a homogeneous population of DG (dominant hemisphere at the first surgical brain mapping), with a consistent preoperative assessment (extensive preoperative cognitive and language assessment, qualitative and quantitative radiological features) and a consistent method of intraoperative registration eloquent spots.

The second limitation is the marginal role of tractography. Even though tractography was present in all the cases as an integral part of the preoperative assessment, we detected inconsistent results and we decided to not use the results for this study due to the limited spatial resolution of the FACT algorithm used in this cohort. Compared with other techniques, our tractographic reconstructions displayed limited peritumoral spatial accuracy (in case of large tumor volume, peritumoral edema) and limitations in reconstructing kissing-crossing fibers in the periventricular and corona radiata region. Aware of these limitations, we routinely included a preoperative tractography in our study for a preoperative anatomy-oriented discussion. We believe that using DTI results as complementary anatomical information provides us with a better surgical planning with possible predictions of functional limits of our resection [38,80]. We agree with other authors encouraging the research and educational role of DTI but its intrinsic limitations (at least with FACT algorithm) affect the clinical use in glioma patients [37,38,80].

Third, the use of neuronavigation to acquire and analyze eloquent spots may raise some criticism because of the possible brain-shift effect. First, we acquired the eloquent spots when the tumor tissue was only disconnected but not removed to avoid this problem. Second, the intraoperative pictures of the surgical field were used as a control to navigation to confirm the exact location of the eloquent spots as described by other authors [64,81].

Fourth, this study of the pattern of tumor-induced plasticity is based on the assumption that the direct electrostimulation effect in a tumoral/peritumoral tissue constitutes evidence of plasticity. However, it should be considered that there is inter-individual variability in the site of functional epicenters (especially cortical) and that the absence of a functional response to direct electrostimulation might correspond to a false negative in some cases. For this reason, our results should be carefully interpreted.

Fifth, we decided to not include in the analysis the eloquent spots acquired outside the peritumoral area. Since we used for instance speech arrest to detect the level of stimulation despite the location of the tumors independently of tumor location, not all the mapped eloquent spots were in functional relationship with the tumors. Then, since no mapping of the interhemispheric/bi-hemispheric adaptive mechanisms was performed with TMS or fMRI, we decided to only focus on tumoral/peritumoral eloquent spots to link focal-local phenomena of adaptation to the preoperative assessment.

Finally, the number of the domains mapped intraoperatively was not as extensive as the preoperative assessment. Although a number of critical brain processes were carefully selected, technical constraints (related to direct electrostimulation) and the clinical context (limited time) prevented us from assessing multiple and important high-level processes (emotions, face recognition, etc.); this may have caused us to overestimate the degree of plasticity at the peritumoral level.

## 5. Conclusions

DG displayed a pattern of early cortical neuroplasticity, shifting the functions to adjacent cortices. This adaptive mechanism seems exhausted at the moment of diagnosis with high risk of finding of intratumoral subcortical eloquent spots. Age may represent an important factor to predict the adaptive mechanisms of neuroplasticity, but at the individual level the prediction of resection grade and eloquent spots seems not possible. An extensive preoperative neuropsychological and language assessment is important to define the involvement of large-scale networks for cognitive functions and detect signs of tumor-induced neuroplasticity. A systematic use of advanced neuroimaging techniques including topography and white matter infiltration analysis is necessary to identify less compensable areas and their link with epileptic onset, NPS and language impairment. A more systematic integration of functional and radiological assessment before awake surgery may lead to a better comprehension of the connectomic brain organization at the individual scale and therefore to a better oncological/functional balance.

## Figures and Tables

**Table 1:** Summary of the descriptive results of demographic, radiological, histological, intraoperative and outcome variables.

Type of Variables		Values
Demographic variables		
Age	mean (SD)	40.36 (10.8)
Gender	m (%)/f (%)	25 (69.4)/11(30.6)
Radiological variables		
Tumor volume	mean (SD)	57.30 (47.4)
Tumor border	sharp (%)/diffuse (%)	14 (38.9)/22(61.1)
Brain-Grid voxels	median (IQR)	6 (4–8)
Clinical variables		
Onset symptoms	*n* (%)	
EP focal		15(41.7)
Ep generalized		11(30.6)
Headache		1 (2.8)
Incidental		9 (25.0)
Preoperative language imp.	y (%)/*n* (%)	24 (66.7)/12 (33.3)
Preoperative NPS imp.* * only in 26 patients	y (%)/*n* (%)	22 (84.6)/4 (15.4)
Histo-pathological variables		
Histology	*n* (%)	
Astrocytomas		23 (63.9)
Oligodendrogliomas		13 (36.1)
Grade	*n* (%)	
A2		12 (33.3)
A3		11 (30.6)
O2		4 (11.1)
O3		9 (25.0)
IDH 1-2 status	(m/NOS)	
A2		8/4
A3		9/2
O2		2/2
O3		7/2
Surgical variables		
Eloquent tumor	y (%)/*n* (%)	27 (75)/9 (25)
Intra-tumoral spots cortical.	mean (SD)	0.36 (0.93)
Intra-tumoral spots Subcortical	mean (SD)	1.33 (1.37)
Peritumoral spots cortical	mean (SD)	1.39 (1.47)
Peritumoral subcortical	mean (SD)	1.00 (1.37)
Intra-tumoral spots	mean (SD)	1.61 (1.69)
Peritumoral spots	mean (SD)	2.36 (2.1)
Cortical spots total	mean (SD)	4.33 (2.7)
Subcortical spots total	mean (SD)	2.72 (2.17)
Resection grade	mean (SD)	79.07 (15.8)
Outcome variables		
Survival	years (SD)	3.36 (1.8)

m, male; f, females; SD, standard deviation; IQR, inter quartile range; No., number of cases; EP, epileptic onset; imp, impairment; NPS, neuropsychological; IDH1-2 status m, mutant; NOS, not otherwise specified according to WHO-2016 classification system [58]. * The Neuropsychological assessment was performed only in 26 patients.

**Table 2:** A summary of the intraoperative eloquent spots displayed for each patient and organized according to the proximity to the FLAIR signal. Spots acquired within the FLAIR tumor signal were considered intratumoral (red color shading, cortical or subcortical). Spots acquired within 1–10 mm from the tumor border were included as peritumoral (orange color shading, cortical or subcortical). Those spots acquired with a distance superior to 10 mm were considered outside the tumor border (green color shading, cortical or subcortical).

Pat N	Localization	Intratumoral		Peritumoral		Outside Tumor Border		Total Number	
		C	SC	C	SC	C	SC	C	SC
**1**	F-o L	SO			SO			1	1
**2**	F L	SO	SA		SO			1	2
**3**	F-T-I L		Hand (M)			SO Face (M)		2	1
**4**	F-T-I L				Arm (M)	SOx2		2	1
**5**	F-I L		SP SP	SO SA		SO		3	2
**6**	T-I L			SA SO		SO x3 Mouth (M)		6	0
**7**	F-T-I L		An	SO SA		SO Mouth (M)		4	1
**8**	F-T-I L					SA, Mouth (M)		2	0
**9**	F-I L		SP			SO, Mouth (M)		2	1
**10**	F L			SOx2 Mouth (M)	SAx2	Hand (M) Face (M) Mouth (M)x2 Tongue (M) An x2 SA		11	2
**11**	T-I L			SO		SOx2 Hand (M) SA		5	0
**12**	DLPFC L			SA	SA Mc	Hand (M)x3 PP SA		6	2
**13**	F-T-I L	SO	SP SA Mouth (M)	Mouth (M) Tongue (M)		SP SAx2 An		6	3
**14**	F-T-I L	An	SP SA			SOx2 Face (M) An		5	2
**15**	F-I L		SA	SAx2		SO Mouth (M) SAx2		6	1
**16**	DLPFC R		Mc	Hand (M)		Hand (M) Arm (M) Tongue (M)		4	1
**17**	F o L			SOx3 SAx3	SA Sox2	Face (M) Mouth (M)x2 SO SA		11	3
**18**	P L		WMx3	Hand (S) Arm (S)	Arm (S)	Leg (M) Arm (M)		4	4
**19**	T-P-O L		Anx3 Vifx2 Mouth (M)	Anx2		Tongue (S)		3	6
**20**	F-I L		An Mouth (M) SO	Tongue (M) Mouth (M) SOx2		Tongue (M)x2 Mouth (M)		7	3
**21**	P L		SAx2 Mouth (S)	SO Mouth (M) Tongue (S)			Mouth (M) Hand (M)	3	5
**22**	F-T-I L	SO SP	SP			Mouth (M)x2 Face (M) Tongue (M)x2 SAx2 PPx2		11	1
**23**	T-P-I L	SAX2 SO Aux2	SP		SP	Mouth (M) SA PP	SP	8	3
**24**	T-O L		R	Anx2 SA	SPx2	SA	VifX2	4	5
**25**	F L			An	WM SA	Ha (M) SA		3	2
**26**	F-I L		SAx2 SP	SA Tongue (M) SO SA	VA SA	SOx2	Arm (M) Hand (M) Face (M)	6	8
**27**	F L		SP		Anx3 SA SP	SO		1	6
**28**	T-I-O R					SO Face (M)		2	0
**29**	T-I L		PPx2		Vis PPx2	SO Face (M)		2	5
**30**	T-I L		SP		SP Vis	SO		1	3
**31**	T-I L		SP	SP		SOx3	Arm (M) SO	4	3
**32**	P L		SA	Hand (M) SPP	An x2	Face (M)		3	3
**33**	F-T-I L		PPx2 An Mc	PPx2 Mc		SOx2		5	4
**34**	P L	Mouth (S)		SA Mouth (M)	PP SAx4	SOx2 Mouth (M)	SAx2 SO Vis	6	9
**35**	P L		SPPx2	SPP	SP	SO SA		3	3
**36**	SMA L	Mc	Mc, AN			SO	Hand (M)	2	3

Locations: F, frontal; P, Parietal; T, temporal; I, insular; O, occipital; o, opercular; SMA, supplementary motor area; DLPFC, Dorso lateral prefrontal cortex; L, left side; R, right side. M, Motor; S, Sensory; SP, Semantic Paraphasia; Mc, Motor control (including negative motor mapping); SPP, spatial perception; An, Anomia; SA, speech articulation domain (including verbal apraxia, latency, dysarthria); SO, speech output domain (Including Speech arrest); VIS, Visceral sensation; Au, Auditory phenomena; R, reading; Vif, Visual field; Wm, working memory.

**Table 3:** Summary of the Spearman’s correlation analysis for numeric and continuous variables. The distribution of intraoperative eloquent spots was not normally distributed (Shapiro–Wilk test *p* < 0.001) and therefore a nonparametric test was chosen for the correlation analysis.

Variables		Age	Tumor Volume	Brain-Grid Voxels	Intratumoral Cortical	Intratumoral Subcortical	Peritumoral Cortical	Peritumoral Subcortical	Resection Grade
Age	Corr. Co	1	0.161	0.101	0.406 *	−0.161	−0.451 *	−0.089	−0.127
	*p*		0.349	0.557	0.014	0.399	0.006	0.605	0.462
Tumor volume	Corr. Co	0.161	1	0.689 *	0.17	0.094	−0.226	−0.511 *	−0.627 *
	*p*	0.349		0	0.32	0.587	0.184	0.001	0
Brain-Grid voxels	Corr. Co	0.101	0.689 *	1	0.012	0.155	−0.112	−0.326	−0.316
	*p*	0.557	0		0.944	0.366	0.517	0.052	0.06
Intratumoral eloquent spots cortical	Corr. Co	0.406 *	0.17	0.012	1	0.024	−0.366 *	−0.037	−0.411 *
	*p*	0.014	0.32	0.944		0.891	0.028	0.828	0.013
Intratumoral eloquent spots Subcortical	Corr. Co	−0.145	0.094	0.155	0.024	1	0.264	−0.281	−0.122
	*p*	0.399	0.587	0.366	0.891		0.119	0.097	0.477
Peritumoral eloquent spots cortical	Corr. Co	−0.451 *	−0.226	−0.112	−0.366 *	0.264	1	0.068	0.153
	*p*	0.006	0.184	0.517	0.028	0.119		0.695	0.372
Peritumoral eloquent spots subcortical	Corr. Co	−0.089	−0.511 *	−0.326	−0.037	−0.281	0.068	1	0.501 *
	*p*	0.605	0.001	0.052	0.828	0.097	0.695		0.002
Resection grade	Corr. Co	−0.127	−0.627 *	−0.316	−0.411 *	−0.122	0.153	0.501 *	1
	*p*	0.462	0	0.06	0.013	0.477	0.372	0.002	

Corr. Co, correlation coefficient. * Correlation is significant with *p* value < 0.05.

**Table 4:** Summary of the correlation between categorical and dichotomous variables with Pearson’s chi square analysis and contingency coefficient.

Variables	Correlation Analysis		
	X^2^		
	*p*	Coefficient	Strength/Direction
Eloquent tumors/Age cut-off	0.439	0.128	
Eloquent tumors/Gender	0.531	0.104	
Eloquent tumors/Tumor volume cut-off	1.00	0.000	
Eloquent tumors/Radiological border	0.693	0.066	
Eloquent tumors/BG voxels cut-off	0.654	0.074	
Eloquent tumors/A3C2S2	0.197	0.210	
Eloquent tumors/A4C1S2	0.700	0.064	
Eloquent tumors/A4C2S2	0.808	0.040	
Epilepsy/Age cut-off	0.739	0.055	
Epilepsy/Gender	0.446	0.126	
Epilepsy/Tumor volume cut-off	0.001 *	0.494	Moderate/+
Epilepsy/Radiological border	0.497	0.112	
Epilepsy/BG voxels cut-off	0.244	191	
Epilepsy/A3C2S2	0.007 *	0.407	Moderate/+
Epilepsy/A4C1S2	0.457	0.123	
Epilepsy/A4C2S2	0.000 *	0.536	High/+
NPS impairment/Age cut-off	0.208	0.240	
NPS impairment/Gender	0.102	0.305	
NPS impairment/Tumor volume cut-off	0.356	0.178	
NPS impairment/Radiological border	0.150	0.272	
NPS impairment/BG voxels cut-off	0.019 *	0.418	Moderate/+
NPS impairment/A3C2S2	0.482	0.137	
NPS impairment/A4C1S2	0.054	0.307	
NPS impairment/A4C2S2	0.187	0.251	
Language impairment/Age cut-off	0.058	0.302	
Language impairment/Gender	0.798	0.043	
Language impairment/Tumor volume cu-off	0.058	0.302	
Language impairment/Radiological border	0.091	0.271	
Language impairment/BG voxels cut-off	0.236	0.194	
Language impairment/A3C2S2	0.188	0.214	
Language impairment/A4C1S2	0.157	0.229	
Language impairment/A4C2S2	0.766	0.050	
Eloquent tumors/Epilepsy	0.667	0.071	
Eloquent tumors/NPS impairment	0.562	0.113	
Eloquent tumors/Language impairment	1.00	0.000	
Epilepsy/NPS impairment	0.114	0.296	
Epilepsy/Language impairment	0.792	0.044	
NPS impairment/Language impairment	0.065	0.340	

* Correlation is significant with *p* value < 0.05. Strength and direction of correlation (positive or negative) are indicated for the significant ones.

**Table 5:** Summary of the logistic regression analysis. Univariate model with NPS impairment, epilepsy and language impairment as dependent variables correlated with the number of intraoperative eloquent spots and the different infiltrated BG voxels. The lower part shows the result from the multivariate analysis with Eloquent tumors as dependent variable and all the demographic, radiological and clinical variables analyzed as predictor factors. * Correlation is significant with *p* value < 0.05. HR, Hazard Risk; CI, confidence interval.

Variables	Binary Logistic Regression		
Univariate	*p*	HR	CI (95%)
NPS impairment/intratumoral eloquent spots cortical	0.201	3.679	0.501–27.036
NPS impairment/intratumoral eloquent spots subcortical	0.019 *	2.200	1.140–4.244
NPS impairment/peritumoral eloquent spots cortical	0.096	1.464	0.935–2.294
NPS impairment/peritumoral eloquent spots subcortical	0.112	1.548	0.903–2.651
Epilepsy/intratumoral eloquent spots cortical	0.105	5.429	0.704–41.875
Epilepsy/intratumoral eloquent spots subcortical	0.028 *	1.766	1.064–2.929
Epilepsy/peritumoral eloquent spots cortical	0.047 *	1.533	1.069–2.337
Epilepsy/peritumoral eloquent spots subcortical	0.251	1.288	0.836–1.985
Language impairment/Intratumoral eloquent spots cortical	0.196	2.554	0.616–10.583
Language impairment/Intratumoral eloquent spots subcortical	0.098	1.421	0.937–2.153
Language impairment/Peritumoral eloquent spots cortical	0.511	1.118	0.802–1.558
Language impairment/Peritumoral eloquent spots subcortical	0.693	0.924	0.624–1.368
NPS impairment/A3C2S2 infiltration	0.007 *	7.500	1.715–32.796
NPS impairment/A4C1S2 infiltration	0.121	2.500	0.784–7.971
NPS impairment/A4C2S2 infiltration	0.019 *	3.750	1.245–11.299
Epilepsy/A3C2S2 infiltration	0.002 *	5.500	1.895–15.960
Epilepsy/A4C1S2 infiltration	0.127	3.500	1.152–10.633
Epilepsy/A4C2S2 infiltration	0.001 *	6.250	2.175–17.958
Language impairment/A3C2S2 infiltration	0.024 *	2.714	1.141–6.457
Language impairment/A4C1S2 infiltration	0.638	1.250	0.493–3.167
Language impairment/A4C2S2 infiltration	0.100	1.900	0.883–4.086
**Multivariate**			
Eloquent tumors/Preoperative NPS impairment	0.003 *	6.333	1.874–21.402

## Data Availability

The data that support the findings of this study are available on request from the corresponding author. The data are not publicly available due to privacy and ethical restrictions.

## Supplementary Materials

The following files are available online at https://www.mdpi.com/2077-0383/10/5/1108/s1. File S1: Summary of the neuropsychological and language tests administered pre- and postoperatively in patients with low-grade gliomas.
