# Supplementary material for: Role of Preoperative Assessment in Predicting Tumor-Induced Plasticity in Patients with Diffuse Gliomas
Source: J Clin Med. 2021 Mar 7;10(5):1108. doi: 10.3390/jcm10051108 (PMC7961995; doi:10.3390/jcm10051108)
Supplement: Supplementary file 1 [file jcm-10-01108-s001.pdf]

Electronic Supplementary material 1

Neuropsychological tests	Speech/Language tests
Digit Span[1]	BeSS[9]
Coding[2]	Boston Naming Test (BNT)[10]
Trail Making Test (TMT) trials 1-5[3]	Token test (version C)[11]
Rey Auditory Verbal Learning Test (RAVLT) Swedish version [4]	Neurolingvistisk afasiundersökning (A-ning)[12]
Brief Visuospatial Memory Test-Revised (BVMT-R)[5]	Word fluency (FAS, Animals and Verbs)[13] LS
Rey Complex Figure Test (RCFT)[6]	Reading Words and Nonwords[14]
Zoo Map Test[7]	Oralmotorisk diadochokinesi[15]
Color-Word Interference Test trials 1-4[3]	STAVUX[16]
Hospital Anxiety and Depression Scale (HADS)[8]	

Summary of the neuropsychological and language tests administered pre- and postoperatively in patients with low-grade gliomas.

References

1) Wechsler, D. (2008). Wechsler Adult Intelligence Scale—Fourth Edition. San Antonio, TX: Pearson

2) Randolph, C. (1998). RBANS Manual: Repeatable battery for the assessment of neuropsychological status. San Antonio, TX: The Psychological Corporation.

3) Delis, D.C., Kaplan, F. & Kramer, J.H. (2001). Delis-Kaplan executive function system. San Antonio, TX: The Psychological Corporation.

4) Schmidt, M. (1996) *Rey Auditory and Verbal Learning Test. A Handbook.* Los Angeles: Western Psychological Services.

5) Benedict, R.H.B. (1997). *The Brief Visual Memory Test-Revised.* Lutz, FL: Psychological Assessment Resources

6) Meyers, J.E. & Meyers, K.R. (1995) *Rey Complex Figure Test and Recognition Trial.* Lutz, FL: Psychological Assessment Resources.

7) Wilson, B.A., Alderman, N., Burgess, P.W., et al. (1996*). Behavioural assessment of the dysexecutive syndrome.* Bury St. Edmunds, UK: Thames Valley Test. 8) Zigmond, A.S., & Snaith, R.P. (1983). The Hospital Anxiety and Depression Scale. *Acta Psychiatrica Scandinavica*, 67, 361-370.

9) Laakso, K., Brunnegård, K., Hartelius, L., & Ahlsén, E. (2000). Assessing high-level language in individuals with multiple sclerosis: A pilot study. Clinical Linguistics & Phonetics, 14(5), 329–349.

10) Kaplan, E., Goodglass, H., & Weintraub, S. (1983). Boston Naming Test. 11) De Renzi, E., & Vignolo, L. A. (1962). The Token test: A sensitive test to detect receptive disturbances in aphasia. Brain, 85, 665-678.

Svensk normering av: Apt, P. (2008). Token test. Malmö: MAS.

12) Werner, C. & Lindström, E. (1995). A-ning. Neurolingvistisk afasiundersökning. Stockholm: Ersta diakonisällskap.

13) Spreen, O. & Benton, A. L. (1969). Neurosensory Center Comprehensive Examination for Aphasia. Victoria, Canada: University of Victoria.

14)Johansson, M-G. (2004). LS. Stockholm: Psykologiförlaget AB.

15) Hartelius, L. (2015). Dysartri - bedömning och intervention. Lund: Studentlitteratur AB.

16) Nilsson, E. & Pettersson, K. (2009). Utveckling och normering av ett stavningstest för vuxna (STAVUX). Magisteruppsats i logopedi. Institutionen för neurovetenskap. Uppsala Universitet.
